# Endophytic Mycoflora and Their Bioactive Compounds from *Azadirachta Indica*: A Comprehensive Review

**DOI:** 10.3390/jof4020042

**Published:** 2018-03-24

**Authors:** Eyob Chukalo Chutulo, Raju Krishna Chalannavar

**Affiliations:** 1 Department of Biology, Wolaita Sodo University, Wolaita Sodo 138, Ethiopia; eyob.chukalo@wsu.edu.et; 2 Department of Biosciences, Mangalore University, Mangalagangothri 574199, India; 3 Department of Applied Botany, Mangalore University, Mangalagangothri 574199, India

**Keywords:** endophytic fungi, plant tissues, bioactive compounds, biological activities

## Abstract

Plants are all inhabited by endophytic fungi in the interior of their tissues. The neem tree *Azadirachta* is an Indian lilac used for various therapeutic purposes in different forms of preparations. This plant hosts different types of endophytic fungi. In some cases, different tissues of a given plant are inhabited by different endophytic fungi which are discussed in this paper. Recently, there have been new reports on endophytic fungi and their bioactive compounds from *Azadirachta indica*. The biological function of bioactive compounds was discussed in view of their future industrial prospects. There are a number of different research investigations that examine the endophytes isolated and screened for their potential bioactive secondary metabolites from neem, but there is no comprehensive review on neem endophytes and their secondary metabolites to bring all trends from different researchers together. Therefore, in this review, we have discussed the endophytic fungi from the different tissues of neem, in view of the latest understandings of antimicrobial, antioxidant, and pathogenicity target compounds. Importantly, tracing the previous findings would pave the way to forecast the missing link for future work by researchers.

## 1. Introduction

The term “endophyte” refers to all microorganisms that colonize internal plant tissues for all or part of their lifetime [1]. They cause unapparent and asymptomatic infection and live entirely within plant tissues. They cause no symptoms of disease [2]. Endophytes are the chemical synthesizers inside plants and plants have been extensively investigated for their endophytic microbial complement [3].

Endophytes are an under-investigated group of microorganisms that represent a plentiful and renewable source of bioactive and chemically new compounds with potential for exploitation in a wide variety of medical, agricultural, and industrial realms [4]. They are a taxonomically and ecologically heterogeneous group of organisms; mainly belonging to Ascomycota, coelomycetes, and hyphomycetes [5,6,7,8].

All plants are inhabited internally by diverse microbial communities comprising bacteria, archaea, fungi, and protista [1,9]. The association between fungal endophytes and their host plant is due to the result of unique adaptations which enable the endophytes to harmonize their growth with that of their host [10]. These endophytic communities are accountable to either partial or complete biosynthesis of host plant secondary metabolites [8,11].

Endophytic biotechnology can be used for the efficient production of agriculturally, industrially and economically important plants and plant products. The rational application of endophytes to manipulate the microbiota, intimately associated with plants, can help in enhancement of production of the agricultural product, increased production of key metabolites in medicinal and aromatic plants, as well as adaption to new bio-geographic regions through tolerance to various biotic and abiotic conditions [12].

Endophytes have recently generated significant interest in the microbial chemistry community due to their great potential to contribute to the discovery of new bioactive compounds. It has been suggested that the close biological association between endophytes and their plant host results in the production of a great number and diversity of biologically active molecules compared to epiphytes or soil-related microbes [13]. Moreover, the symbiotic nature of this relationship indicates that endophytic bioactive compounds are less toxic to the cell, as these chemicals do not kill the eukaryotic host system. This is particularly important to the medical community as potential drugs may not adversely affect human cells [14].

*Azadirachta indica* is widely used for the investigation of endophytes and their secondary metabolites, but it remains crucial to further extend the study as it is one of the major traditional medicinal plants used by about 80% of developing nations. This is because the discovery of new bioactive compounds as well as new endophytic fungi from *Azadirachta indica* has been reported recently. Hence, this review discusses the latest research advances on endophytic fungi from the neem tree, including types of endophytic fungi identified, described, bioactive compounds, and biological activities of endophytes to trace the current scope of knowledge of endophytes. It gives collective recent knowledge on antimicrobial activities of endophytic fungi of neem and on recent advances concerning their efficacy as potential antimicrobials to fight the current health problems due to emerging multidrug-resistant pathogens. It also gives focus to the well-studied endophytic sources of potential antimicrobials with emphasis on their future prospects of endophytes on human pathogens based on the recent research outputs.

The chance of getting new bioactive compounds from endophytes depends on several factors, for example, culture condition, tissue type, and age of host, the degree of interaction of endophytes with their photobionts in nature, the genetic basis of mycobionts and photobionts. Leaving aside most of these factors, by modifying the culture condition different investigators are reporting new endophytic isolates [15] and new bioactive compounds in recent studies. This signifies the continuous nature of exploiting the endophytes even from largely exploited hosts as well. Importantly, to our knowledge, there is no review on the endophytic fungi and their bioactive compounds from neem plants. Therefore, the need to review the endophytic fungi and their bioactive compound from *Azadirachta indica* is thus apparent. Ultimately, we believe that this review provides relevant research trends on the subject.

### 1.1. Plant-Endophyte Metabolism

Plant and endophyte are under continuous interaction in nature in their symbiotic existence. Their metabolism can interact on many levels: (a) the endophyte induces host metabolism, (b) the host induces endophyte metabolism, (c) host and endophyte share parts of a specific pathway and contribute partially, (d) the host can metabolize products from the endophyte and vice versa, (e) the endophyte can metabolize secondary compounds from the host. The two latter possibilities can be accomplished by only one, several or all enzymatic steps for biochemical transformation [11].

Endophytes can influence their host plant’s metabolism, but one can speculate that the established host range could also alter or influence the pattern of secondary metabolites in endophytic fungi. Moreover, the host plants can influence the metabolite pattern in pathogenic fungi [11].

### 1.2. Effects of Endophytes on Neem Plants

Endophytes form a symbiotic relationship with their plant host. It is believed that in many cases the microbes function as the biological defense for the plant against foreign phytopathogens due to the fact that the majority of secondary metabolites occurring in endophytic microorganisms have antimicrobial activity and these have been implicated in protecting the host plant against [16]. The protection mechanism of the endophytes are exerted directly, by releasing metabolites to attack any antagonists or lyse affected cells, and indirectly, by either inducing host defense mechanisms or promoting growth [14]. Antibiotics or hydrolytic enzymes can be released by endophytes to prevent colonization of microbial plant pathogens [13,17].

## 2. Endophytic Fungi from *Azadirachta indica*

Different tissue samples of a given plant in several locations yield the greatest species diversity of endophytes. *Periconia*, *Stenella*, and *Drechslera* were endophytes reported from neem plant [6]. Moreover, the authors of [6] reported *Phomopsis oblonga*, *Cladosporium cladosporioides*, *Pestalotiopsis* sp., *Trichoderma* sp., and *Aspergillus* sp. as the dominant endophytic fungal isolates. The huge and substantial collection of endophytic fungi from neem may represent a peculiar source of the interesting and useful bioactive compounds associated with *Azadirachta indica* such as the azadirachtins and related tetranortriterpenoids [6].

The diversity of endophytic fungi is likely determined by the age of the plant parts [18]. Some endophytes are isolated only from certain specific locations irrespective of the difference in tissue specificity [19]. Therefore, in this section, endophytic fungi from different tissues of *Azadirachta indica* are reviewed and presented in Table 1.

### 2.1. Endophytic Fungi of Leaf

The leaf is an ideal tissue for exploring the endophytes and their secondary metabolites. The leaf has shown the highest report on species richness and colonization of endophytes [6]. The authors of [6] reported the occurrence of five fungal endophytes from asymptomatic green and senescent leaves of neem plant. From these, four were sterile forms and one was *Fusarium avenaceum*. The frequency of occurrence of endophytes was significantly higher in the basal leaflets than in the apical or middle leaflets and in the main vein of the leaflet than in the lamina tissue. The colonization frequency of green leaves by endophytes increased during the rainy season although no new endophyte species could be recovered by their study. The antifungal metabolites present in the leaves could be accounted for by the restricted number of endophytic fungal genera and the absence of common endophytic fungi in the neem leaves. The occurrence of foliar endophytes in tropical trees is influenced by environment, types and chemistry of the host tissue [20], and the nature of interaction between the mycobiont and phycobiont. They have isolated and recorded *Fusarium avenaceum* with some other sterile mycelia. Another study conducted by [21] isolated four endophytic fungal species (*Trichoderma* sp., *Colletotrichum* sp., *Curvularia* sp. and *Chaetomium* sp.) and *Alternaria alternata* [22] from the leaves of *Azadirachta indica* A. Juss.

In most studies of endophyte fungi, the sterile mycelia fungi are reported as mycelia-sterile. Unlike those, the three mycelia-sterile isolated were identified as *Fusarium solani* and *Chaetomium globosum*. Additionally, leaf isolated mycelia-sterile was identified as *F*. *solani* (soil fungus) by Verma [7], and it shows the vertical traveling nature of this fungus from root to upper tissues of the host by the same study.

Tenguria and Khan [23] have isolated 85 endophytic fungi of 10 genera from 200 segments of fresh *Azadirachta indica* leaves. These are *Chaetomium globosum*, *Pestalotiopsis* spp., *Phoma* sp., *Aspergillus flavus*, *Aspergillus niger*, *Alternaria alternata* (Fr.) Keissl., *Fusarium* spp., *Penicillium* spp., *Trichoderma* spp., and Sterile mycelia. Their isolates are almost similar to fungi reported by Taware & Rajurkar [24]. The endophyte *Geotrichum* sp. AL4 was cultivated from the leaf of *Azadirachta indica* and produced novel active components with nematicidal activities [25]. Abubakar and Ndana [26] have isolated five species of endophytic fungi from leaf tissue of *Azadirachta indica* with *Cladosporium* spp. dominated the leaves with the frequency of colonization of 11.3%.

### 2.2. Endophytic Fungi of Stem

The endophytic fungus *Xylaria* sp. YM 311647 was isolated from stems of healthy *Azadirachta indica* collected in China [27]. Another endophytic fungus *Phomopsis* sp. YM 311483 was isolated from surface-sterilized fresh stems of an apparently healthy *Azadirachta indica* specimen collected in China. This fungus produced two new ten-membered lactones which have antifungal potential against plant pathogenic fungi [28].

Wu et al. [29] have isolated and reported the fungal genus *Xylaria* sp. YC-10 from stems of *Azadirachta indica* A. Juss. Abubakar and Ndana [26] have isolated seven species of endophytic fungi from stems (twig) tissue of *A. indica* with *Cryptococcus* spp. reported being the most dominant isolate with high percentage frequency of colonization 13.9%.

### 2.3. Endophytes of Fruit

The endophytic fungi from *A. indica* are mostly known from leaves, bark, and stems but it is uncommon from fruits of this host. Verma et al. [7] have isolated endophytic fungi from unripe fruit and roots. They have isolated 105 endophytic fungi from these unripe fruits, from 29 taxa, at a rate of 68.0%. Their isolates were composed of 11.06% mycelia sterilia, 7.25% coelomycetes, and 81.69% hyphomycetes, while *Humicola*, *Drechslera,* and *Colletotrichum* sp. were obtained exclusively from fruit samples.

### 2.4. Endophytic Fungi of Bark

Mahesh et al. [5] have investigated the endophytic fungi from the inner bark of *A. indica*. They have isolated 77 endophytic fungal isolates belonging to 15 genera composed of hyphomycetes, coelomycetes, ascomycetes and sterile mycelia (Table 1). The endophytes *Curvularia*, *Cochlonema*, *Gliomastix* and *Verticillium* spp. were reported in their study. Most importantly, *Trichoderma*, *Penicillium* and *Pestalotiopsis* spp. were the most dominant endophytes recorded. Verma et al. [6] have isolated and characterized 18 different taxa of endophytic fungi from leaf, stem, and bark of *Azadirachta indica* A. Juss. Their isolates are composed of 62.2% hyphomycetes, coelomycetes (27.4%) and mycelia sterilia (7.7%). Importantly, the endophytic fungi occur more often in leaf segments than stem and bark tissues [6].

Tejesvi et al. [19] have isolated the endophytic fungi *Gliomastix*, *Curvularia*, *Phoma eupyrena*, and *Phyllosticta* from the inner bark of *A. indica*. In another investigation, they have reported three isolates of *Pestalotiopsis microspora* and two isolates of *Bartalinia robillardoides* from the inner bark of *A. indica* [30].

### 2.5. Endophytic Fungi of Root

The endophytic fungus *Chloridium* sp. was isolated from the roots of *A*. *indica* A. Juss. in Varanasi, India. The fungus produces a highly functionalized javanicin, with promising antibacterial activity [31] that might prevent its host from invasion by pathogenic microorganisms.

Verma et al. [7] have isolated 167 endophytic fungi from roots, at a rate of 68.0%. Their isolates are identified into 29 taxa; those composed of mycelia sterilia accounted for 11.06%, coelomycetes 7.25%, while hyphomycetes showed the maximum number of representative isolates (81.69%). They have recorded species such as *Chaetomium globosum*, *Chloridium*, *Scytalidium*, *Nigrospora* and *Verticillium* exclusively from the root. Kusari et al. [32] have reported the isolation and characterization of a novel endophytic fungus *Eupenicillium parvum* in their study.

### 2.6. Endophytic Fungi of Seed and Twigs

Although endophytic fungi isolated from different tissue of plants in several investigations, the reports of endophytic fungi from twigs of neem plant, unlike from other plants, are very few. Ashkezari and Fotouhifar [15] have isolated nineteen new endophytic fungi from a common yew plant. The pathogenic endophyte *Phomopsis azadirachtae*, the cause of devastating die-back disease, were reported from seeds and twigs of neem plants [33]. It is important to note that the twigs of *Azadirachta indica* are a poorly exploited surface for investigation of endophytes. Therefore, it is imperative to conduct further study on the endophytic fungi and their bioactive prospects in the search for potential applications. 

## 3. Current Trends on Bioactive Metabolites from Neem Endophytic Fungi

The production of antimicrobial bioactive compounds by endophytes is currently receiving urgent concern due to the emergence of multidrug-resistant pathogens in chemotherapy. Unlike the chemical synthetics, due to health and environmental issues, there is a strong need of endophytic sources of antimicrobials for their biological and chemical safety effects. There exist many appreciable possibilities for the exploitation of endophytic fungi for the generation of abundant novel biologically active secondary metabolites. Secondary metabolites from microorganisms have proven to be tremendous and enduring sources [47]. The endophytes isolated from the medicinal plants have the potential as an alternative source of these bioactive secondary metabolites [48]. Some of the bioactive compounds produced by endophytic fungi of the neem tree and their biological activities are discussed hereunder.

### 3.1. Melanin Pigment

*Diaporthe* (former name: *phomopsis*) is a phellophytic fungus that lives in the outer layer of the bark [49]. Found in *Azadirachta indica*, it synthesized and deposited DOPA (3,4- dihydroxyphenylalanine) type of melanin on their hyphae. Melanin produced by the endophyte, *Phomopsis*, provides adaptations, which include mechanisms for overcoming host barriers, successful competition with other phylloplane fungi and surviving harsh environmental conditions [50] and in the widespread occurrence of the endophyte [51].

Melanin pigment production is catalyzed by the enzyme tyrosinase produced by endophytic fungi *Fusarium* spp. from *Azadirachta indica*. The fungi produced 2.8 U/mol (L-DOPA) extracellular tyrosinase activity. This enzyme is essential for pigmentation, an important factor in wound healing and primary immune response. It has a great application in environmental, pharmaceuticals, cosmetics and food industries [52].

Even though endophytes are known for their antibacterial, antitumor, immunomodulatory, anti-inflammatory and antiviral activities, there are few reports on endophytes for tyrosinase production from neem plants [52]. However, extracellular production of fungal tyrosinase was reported, firstly, from a filamentous fungus, *Trichoderma reesei* [53]. Therefore, future research should focus on investigating tyrosinase from neem endophytes due to its higher industrial applications.

### 3.2. Antioxidant Metabolites

Kumaresan et al. [21] have screened endophytic fungi, from the leaf, for their antioxidant bioactive compounds. They have investigated the constituents of antioxidants like phenols, tannin, flavonoid, ascorbic acid, and β-carotene. All isolated endophytic fungi studied by their team possessed in vitro antioxidant activities. Similarly, evaluation of secondary metabolites from *Penicillium species* revealed the presence of saponins in addition to those mentioned above [54]. This is one of the very few reports from neem endophytes for their antioxidant activities. Another study by Zhao et al. [55] reported the natural antioxidant cajaninstilbene acid (100.5 ± 9.4 µg/g dry weight of mycelium) from endophytic fungi *F. solani* isolated from pigeon pea. This Fusarium was also found in neem tree as mentioned in Table 1.

### 3.3. Antimicrobial Secondary Metabolites

Wu et al. [56] reported two solanapyrone analogues (solanapyrones N and O) compounds isolated from the fermentation culture of *Nigrospora* sp. YB-141, an endophytic fungus isolated from *Azadirachta indica* A. Juss growing in the tropical region of Southwest China.

Verma et al. [57] have screened and evaluated the efficacy of six fungal endophytic strains from *Azadirachta indica* A. Juss against dermatophytic fungi *Trichophyton* and *Microsporum*. They have reported production of active compounds by their isolates but identification of the compound is not yet reported. Similarly, evaluation of secondary metabolites from *Penicillium species* isolated from the leaf of neem plant showed that they possessed some degree of antibacterial and antifungal activities [54].

Five 10-membered lactones were isolated from the endophytic fungus, *Phomopsis* sp. YM 311483, obtained from the stem of *Azadirachta indica*. Of these, two compounds (8R-acetoxy-5R-hydroxy-7-oxodecan-9-olide and 7R-acetoxymultiplolide A) were newly recorded by Wu et al. [28]. Two compounds identified as 7R,8R-dihydroxy-3,5-decadien-10-olide and 8R-acetoxymultiplolide A are identical to compounds reported by Tan et al. [58]. All the compounds were evaluated for their antifungal activity against seven plant pathogens, *Aspergillus niger*, *Botrytis cinerea*, *Fusarium avenaceum*, *Fusarium moniliforme*, *Helminthosporium maydis*, *Penicillium islandicum*, and *Ophiostoma minus*. Compound 8R-acetoxymultiplolide A showed the most potent antifungal activities with MIC values in the range of 31.25–500 µg/mL. Interestingly, 8R-acetoxymultiplolide A was more potent [58] than 7R-acetoxymultiplolide A even though their structures differed only in the position of the acetoxy substituent [40]. On the contrary, Tan et al. [58] have reported that compounds 7α,8α-Dihydroxy-3,5-decadien-10-olide and 8α-Acetoxymultiplolide A have not shown any antifungal activities against *Candida albicans*. Therefore, further antimicrobial activity bioassay is deemed necessary to reconcile the discrepancy of the report and to utilize the potential of the isolates and their compounds.

Five guaiane sesquiterpenes isolated from the culture broth of endophytic fungus *Xylaria* sp. YM 311647 showed moderate or weak antifungal activities in a broth microdilution assay against five pathogenic fungi, but no obvious inhibitory activities against *Fusarium avenaceum* [27]. Nine oxygenated guaiane-type sesquiterpenes and three isopimarane diterpenes were reported from this fungus, by Tejesvi et al. [30]. Suggested by Wu et al. [59], 18-norisopimarane diterpene and the diterpene sulfate are effective against human pathogenic fungi.

The extracts of *Pestalotiopsis microspora* isolated by Tejesvi et al. [30] demonstrated the potential of antibacterial activities as therapeutic agents against various pathogens tested. The same species from different isolates by the same work demonstrated antioxidant and antihypertensive properties. From this, it is possible to deduce that this isolate needs to be thoroughly investigated for its commercial use as an antioxidant or antihypertensive agent for the treatment of the respective diseases. 

An investigation of the secondary metabolites from *Chloridium* sp. root isolates by Kharwar et al. [31] resulted in the identification of javanicin. This compound was identical in all respects to a previously described naphthaquinone, javanicin. However, its crystallized structure was confirmed by X-ray crystallography. Javanicin was tested for its antimicrobial potential against human and plant pathogens. Tests demonstrated that javanicin had antimicrobial potential against human and plant pathogens. It was either slightly active or not active against fungi such as *Pythium ultimum*, *Phytophthora infestans*, *Botrytis cinerea*, and *Ceratocystis ulmi*, whereas it was active against *C*. *albicans*, *Escherichia coli*, *Bacillus* sp., and *Fusarium oxysporum* at higher MIC values ranging from 20 and 40 µg/mL. Its activity was recorded against *Rhizoctonia solani* and *Verticillium dahliae* at 10 µg/mL while it was active at 5 µg/mL against *Cercospora arachidicola*. The bacteria that were the most sensitive to the javanicin (2 µg/mL) were *P*. *aeruginosa* and *P*. *fluorescens*. On account of this, it may be ideal for compounds, particularly for valuable selective antibiotic formulations for human and plant pathogens [31].

### 3.4. Natural Insecticides

The production of natural insecticides, azadirachtin A and B, from endophytic fungus *Eupenicillium parvum* was reported by Kusari et al. [42]. These compounds are exclusive to the neem tree, *Azadirachta indica* A. Juss, from where they are currently originated. The production of this compound by the fungus is not only in the mycelium but also released into the culture media. The discovery of this azadirachtin-producing endophytic fungus has vast implications for further research from the ecological and biochemical point of view. Another study conducted by Kaur et al. [22] reported antifeedant and toxic activity against tobacco caterpillar *Spodoptera litura* by *Alternaria alternata*. The larvae fed on the diet supplemented with fungal extract significantly reduced the survival and influence the development and reproduction of *S. litura* due to potent toxicity of *A. alternata*. Additionally, the fungus was known for its immunomodulatory effects on *Spodoptera litura* [60].

The genus *Xylaria* sp. YC-10 isolated from stems of *Azadirachta indica* A. Juss. produced eleven compounds from which eight compounds (5-Methylmellein, 5-Carboxylmellein, Hymatoxin C, Hymatoxin D, Halorosellinic acid, Cerebroside C, Cerevisterol and (2S,3S,4R,2′R)-2-(2′-Hydroxytetracosanoylamino)-octadecane-1,3,4-triol) exhibited weak insecticidal activity against *Plutella xylostella* [29].

### 3.5. Nematicides

Li et al. [25] isolated four compounds from the endophytic fungal strain *Geotrichum* sp. AL4, cultivated from the leaves of the neem tree. They reported two compounds, chlorinated oxazinane derivate (1-[(2R*,4S*,5S*)-2-chloro-4-methyl-1,3-oxazinan-5-yl] ethenone) and an epimer of the former (1-[(2R*,4S*,5R*)-2-chloro-4-methyl-1,3-oxazinan-5-yl] ethanone) and two other known compounds. The two compounds reported by their team were assessed for nematicidal activities against the nematodes *Bursaphelenchus xylophilus* and *Panagrellus redivivus*, and showed noticeable bioactivities. Of known compounds, compound 1-(2,4-dihydroxyphenyl)-ethanone is commonly isolated from plants. However, they have reported, for the first time, this compound from a microbial source showing nematicidal activity. Therefore, the *Geotrichum* sp. AL4 can be an ideal source for the formulation of antinematicidal agents.

### 3.6. Antiparasites

Verma et al. [61] have investigated several endophytic fungal strains from *Azadirachta indica* A. Juss and came up with the endophytic fungal strain *Pestalotiopsis* sp. with a significant anticestodal potential against hydatid cysts *Echinococcus granulosus*. The anticestodal activity observed with *Pestalotiopsis* sp. showed promising scolicidal activity of up to 97% mortality within 30 min of incubation. Since this was the first report that dealt with fungal endophytes for the anticestodal potential activities, further studies to reaffirm its anticestodal biopotential activities through optimized culture condition are thus needed.

## 4. Conclusions

Currently, the demand for health services is growing dramatically, particularly in developing countries, due to the emergence of drug resistance by pathogenic microorganisms. Moreover, the highly-increased recurrence of cancer and other infectious diseases makes the situation a greater tragedy. Hence, it is urgently necessary to investigate new bioactive compounds effective against drug-resistant pathogens for the remedy of the aforementioned diseases. Therefore, endophytic fungi, producer of a wide array of secondary bioactive metabolites with their peculiar potential compounds, namely, melanin, antimicrobials, antioxidant, anti-inflammatory, insecticides, nematicides, etc., are the ideal targets. These bioactive compounds have a range of potential to combat etiologic agents of plants and animal’s disease, prevent cell damage due to reactive oxygen species, crop pests, animal pests, and to target pathogenicity traits of pathogenic microbes for necessary remediation.

To date, very few structures of bioactive compounds are characterized and identified from endophytic fungi of neem, but most of the investigations are restricted to the level of fungal identification and bioactivities assay. The different types of Ascomycota, Basidiomycota and Zygomycota endophytes found in the neem tree are phylogenetically synthesized using modern taxonomy from different systematic reviews (Table 1). Some of the endophytic isolates which failed to sporulate, sterile mycelia, are placed to their natural group based on modern taxonomy. Most importantly, the uncultured endophytic fungi from the neem tree were not reported in the literature surveyed so far. As a result, non-culturable endophytes have not been looked for with a metagenomics method. Metagenomics approaches, if used for neem tree, will allow identifying uncultured endophyte fungi that are ignored when isolation and cultivation of endophytes are done before identification. Similarly, too many authors do not make sufficient use of the modern taxonomy based on phylogenetic analysis for identification of their endophytic isolates. The compounds are identified by few investigators and this may be due to a lack of advanced equipment, but possible cooperation with the institutions with the tools are crucial for further discovery.

Even though there are several records on the antioxidant activities of endophytic fungi from different plant hosts, very few endophytes having these effects are known from *Azadirachta indica*. However, this host is widely used for its various biological activities, as antioxidant properties, in different forms of the formulation. Therefore, more work on the antioxidant activities of its endophytes can exert a profound effect on the search for novel antioxidant bioactive compounds.

## Figures and Tables

**Table 1:** Endophytic fungi from different parts of neem plant.

Division (Subdivision)	Class	Subclass	Order	Family	Genus (Former Name)	Identified by	Plant Tissue	References
Ascomycota	Sordariomycetes	Hypocreomycetidae	Hypocreales	Nectriaceae	*Fusarium*	ITS-5.8S rDNA analysis	R,L,S,B,F	[6,7,19,34,35,36]
(Pezizomycotina)				Hypocreaceae	*Trichoderma*	LSU, SSU, TEF and RPB2 sequence data	R,L,S,B,F	[7,36]
					*Gliomastix*	Phylogeny	L,B	[5,6,19,36]
					*Acremonium*	Phylogeny and DNA-based identification	R,B,F	[7,19,37]
			Glomerellales	Plectosphaerellaceae	*Verticillium*	LSU, SSU, TEF and RPB2 sequence data	R	[7,36]
				Glomerellaceae	*Colletotrichum*	ITS-5.8S rDNA analysis	L,F	[7,21]
		Sordariomycetidae	Sordariales	Chaetomiaceae	*Chaetomium*	ITS-5.8S rDNA analysis	R,L,B	[7,19,23]
					*Humicola*	ITS-5.8S rDNA analysis	F	[7,36]
			Chaetospheriales	Chaetosphaeriaceae	*Chloridium*	ITS	R	[7]
		Xylariomycetidae	Xylariales	Xylariaceae	*Xylaria*	Phylogenetic analysis; Molecular clock analysis	S	[29,34,35,36]
				Amphisphaeriaceae	*Pestalotiopsis*	Phylogenetic analysis; Molecular clock analysis	B,L,S	[5,6,23,30,36]
				Bartaliniaceae	*Bartalinia*	ITS rDNA analysis	B	[38,39]
		Diaporthomycetidae	Trichosphaeriales	Trichosphaeriaceae	*Nigrospora*	ITS-5.8S rDNA analysis	R,S,L,B	[6,7,36]
			Diaporthales	Diaporthaceae	*Diaporthe* (*Phomopsis*)	six-gene phylogeny	S,L,B	[6,34,36,40]
	Leotiomycetes				*Scytalidium*	ITS-5.8S rDNA analysis	R	[7]
	Eurotiomycetes				*Aspergillus*	six-gene phylogeny	R,S,L,B,F	[34]
					*Penicillium*	ITS-5.8S rDNA analysis	R,F	[7]
					*Eupenicillium*	five-gene phylogeny	R,S,L,B	[41,42]
	Dothideomycetes		Pleosporales		*Curvularia*	ITS-5.8S rDNA analysis	R,B,F	[6,7,19]
					*Alternaria*	ITS-5.8S rDNA analysis	R,L,F	[6,7]
					*Drechslera*	ITS-5.8S rDNA analysis	B,F	[6,7]
					*Phoma*	Phylogenetic analysis	L	[36]
					*Periconia*	Phylogenetic analysis; Molecular clock analysis	B	[6,36]
			Capnodiales	Davidiellaceae	*Cladosporium*	ITS-5.8S rDNA analysis	R,L,B,F	[7,43,44]
					*Stenella*		B	[6]
			Botryospheriales	Botryosphaeriaceae	*Phyllosticta*	ITS-5.8S rDNA analysis	R,F	[7,39,45]
Basidiomycota (Agaricomycotina)	Tremellomycetes	Tremellomycetidae	Filobasidiales		*Cryptococcus*	six-gene phylogeny	S	[34,46]
Zygomycota (Zoopagomycotina)			Zoopagales	Cochlonemataceae	*Cochlonema*	Phylogeny	B	[5,19,46]
				Mucoraceae	*Cercinella*	ITS-5.8S rDNA analysis	R	[7]

Note: “R”: root; “L”: Leaf; “B”: Bark; “S”: Stem; “F”: Fruit. The endophytes are isolated from the tissue types.

## References

[1] Hardoim P.R., van Overbeek L.S., Berg G., Pirttilä A.M., Compant S., Campisano A., Döring M. (2015). The Hidden World within Plants: Ecological and Evolutionary Considerations for Defining Functioning of Microbial Endophytes. Microbiol. Mol. Biol. Rev..

[2] Wilson D. (1995). Endophyte : The Evolution of a Term, and Clarification of Its Use and Definition. Oikos.

[3] Ramesha A., Srinivas C. (2014). Antimicrobial activity and phytochemical analysis of crude extracts of endophytic fungi isolated from *Plumeria acuminata* L. and *Plumeria obtusifolia* L.. Eur. J. Exp. Biol..

[4] Strobel G., Daisy B., Castillo U., Harper J. (2004). Natural Products from Endophytic Microorganisms. J. Nat. Prod..

[5] Mahesh B., Tejesvi M.V., Nalini M.S., Prakash H.S., Kini K.R., Subbiah V., Shetty H.S. (2005). Endophytic mycoflora of inner bark of *Azadirachata indica*. Curr. Sci..

[6] Verma V.C., Gond S.K., Kumar A., Kharwar R.N., Strobel G. (2007). The endophytic mycoflora of bark, leaf, and stem tissues of *Azadirachta indica* A. Juss (Neem) from Varanasi (India). Microb. Ecol..

[7] Verma V.C., Gond S.K., Kumar A., Kharwar R.N., Boulanger L.A., Strobel G.A. (2011). Endophytic Fungal Flora from Roots and Fruits of an Indian Neem Plant *Azadirachta indica* A. Juss., and Impact of Culture Media on their Isolation. Indian J. Microbiol..

[8] Rajagopal K., Maheswari S., Kathiravan G. (2012). Diversity of endophytic fungi in some tropical medicinal plants—A report. Afr. J. Microbiol. Res..

[9] Verma V.C., Gond S.K., Kumar A., Mishra A., Kharwar R.N., Gange A.C. (2008). Endophytic Actinomycetes from *Azadirachta indica* A. Juss.: Isolation, Diversity, and Anti-microbial Activity. Microb. Ecol..

[10] Verma V.C., Singh S.K., Kharwar R.N. (2012). Histological Investigation of Fungal Endophytes in Healthy Tissues of *Azadirachta indica* A. Juss. Kasetsart J. Nat. Sci..

[11] Ludwig-Müller J. (2015). Plants and endophytes: Equal partners in secondary metabolite production?. Biotechnol. Lett..

[12] Wani Z.A., Ashraf N., Mohiuddin T., Riyaz-Ul-Hassan S. (2015). Plant-endophyte symbiosis, an ecological perspective. Appl. Microbiol. Biotechnol..

[13] Strobel G.A. (2003). Endophytes as sources of bioactive products. Microbes Infect..

[14] Alvin A., Miller K.I., Neilan B.A. (2014). Exploring the potential of endophytes from medicinal plants as sources of antimycobacterial compounds. Microbiol. Res..

[15] Ashkezari S.J., Fotouhifar K.-B. (2017). Diversity of endophytic fungi of common yew (*Taxus baccata* L.) in Iran. Mycol. Prog..

[16] Gunatilaka A.A.L. (2006). Natural Products from Plant-Associated Microorganisms : Distribution, Structural Diversity, Bioactivity, and Implications of Their Occurrence. J. Nat. Prod..

[17] Berg G., Hallmann J., Schulz P.D.B.J.E., Boyle D.C.J.C., Sieber D.T.N. (2006). Control of Plant Pathogenic Fungi with Bacterial Endophytes.

[18] Qi F., Jing T., Zhan Y. (2012). Characterization of Endophytic Fungi from Acer ginnala Maxim. in an Artificial Plantation: Media Effect and Tissue-Dependent Variation. PLoS ONE.

[19] Tejesvi M.V., Mahesh B., Nalini M.S., Prakash H.S., Kini K.R., Subbiah V., Shetty H.S. (2006). Fungal endophyte assemblages from ethnopharmaceutically important medicinal trees. Can. J. Microbiol..

[20] Rajagopal K., Suryanarayanan T.S. (2000). Isolation of endophytic fungi from leaves of neem (*Azadirachta indica* A. Juss.). Curr. Sci..

[21] Kumaresan S., Karthi V., Senthilkumar V., Balakumar B.S., Stephen A. (2015). Biochemical Constituents and Antioxidant Potential of Endophytic Fungi isolated from the Leaves of *Azadirachta indica* A. Juss (Neem) from Chennai. J. Acad. Ind. Res..

[22] Kaur H.P., Singh B., Kaur A., Kaur S. (2013). Antifeedent and toxic activity of endophytic *Alternaria alternata* against tobacco caterpillar *Spodoptera litura*. J. Pest Sci..

[23] Tenguria R.K., Khan F.N. (2011). Distribution of Endophytic Fungi in Leaves of *Azadirachta indica* A. JUSS. (Neem) of Panchmarhi Biosphere Reserve. Curr. Bot..

[24] Taware A.S., Rajurkar S.K. (2015). Diversity assessment of endophytic fungi from *Azadirachta indica* A. Juss. from various regions of Aurangabad, Maharashtra (India). Int. J. Innov. Sci. Eng. Technol..

[25] Li G.H., Yu Z.F., Li X., Wang X.B., Zheng L.J., Zhang K.Q. (2007). Nematicidal metabolites produced by the endophytic fungus *Geotrichum* sp. AL4. Chem. Biodivers..

[26] Abubakar S., Ndana R.W. (2016). Preliminary study of Endomycodiversity among three ethnomedicinal plants from family Meliaceae in Nigeria. J. BioSci. Biotechnol..

[27] Huang R., Xie X.S., Fang X.W., Ma K.X., Wu S.H. (2015). Five new guaiane sesquiterpenes from the endophytic fungus *Xylaria* sp. YM 311647 of *Azadirachta indica*. Chem. Biodivers..

[28] Wu S.H., Chen Y.W., Shao S.C., Wang L.D., Li Z.Y., Yang L.Y., Li S.L., Huang R. (2008). Ten-membered lactones from *Phomopsis* sp., an endophytic fungus of *Azadirachta indica*. J. Nat. Prod..

[29] Wu S.-H., Chen Y.-W., Miao C.-P. (2011). Secondary metabolites of endophytic fungus *Xylaria* sp. YC-10 of *Azadirachta indica*. Chem. Nat. Compd..

[30] Tejesvi M.V., Kini K.R., Prakash H.S., Subbiah V., Shetty H.S. (2008). Antioxidant, antihypertensive, and antibacterial properties of endophytic *Pestalotiopsis* species from medicinal plants. Can. J. Microbiol..

[31] Kharwar R.N., Verma V.C., Kumar A., Gond S.K., Harper J.K., Hess W.M., Lobkovosky E., Ma C., Ren Y., Strobel G.A. (2009). Javanicin, an antibacterial naphthaquinone from an endophytic fungus of neem, *chloridium* sp.. Curr. Microbiol..

[32] Kusari S., Singh S., Jayabaskaran C. (2014). Biotechnological potential of plant-associated endophytic fungi: Hope versus hype. Trends Biotechnol..

[33] Vedashree S., Sateesh M.K., Lakshmeesha T.R., Mohammed S.S. (2013). Screening and assay of extracellular enzymes in *Phomopsis azadirachtae* causing die-back disease of neem. J. Agric. Technol..

[34] James T.Y., Kauff F., Schoch C.L., Matheny P.B., Hofstetter V., Cox C.J., Celio G., Gueidan C., Fraker E., Miadlikowska J. (2006). Reconstructing the early evolution of Fungi using a six-gene phylogeny. Nature.

[35] Hongsanan S., Maharachchikumbura S.S.N., Hyde K.D., Samarakoon M.C., Jeewon R., Zhao Q., Al-Sadi A.M., Bahkali A.H. (2017). An updated phylogeny of Sordariomycetes based on phylogenetic and molecular clock evidence. Fungal Divers..

[36] Maharachchikumbura S.S., Hyde K.D., Jones E.G., McKenzie E.H., Huang S.K., Abdel-Wahab M.A., Daranagama D.A., Dayarathne M., D’souza M.J., Goonasekara I.D. (2015). Towards a natural classification and backbone tree for Sordariomycetes. Fungal Divers..

[37] Summerbell R.C., Gueidan C., Schroers H.J., de Hoog G.S., Starink M., Rosete A.Y., Guarro J., Scott J.A. (2011). Acremonium phylogenetic overview and revision of *Gliomastix*, *Sarocladium*, and *Trichothecium*. Stud. Mycol..

[38] Wijayawardene N.N., Hyde K.D., Wanasinghe D.N., Papizadeh M., Goonasekara I.D., Camporesi E., Bhat D.J., McKenzie E.H.C., Phillips A.J.L., Diederich P. (2016). Taxonomy and phylogeny of dematiaceous coelomycetes. Fungal Divers..

[39] Senanayake I.C., Maharachchikumbura S.S.N., Hyde K.D., Bhat J.D., Jones E.B.G., McKenzie E.H.C., Dai D.Q., Daranagama D.A., Dayarathne M.C., Goonasekara I.D. (2015). Towards unraveling relationships in Xylariomycetidae (Sordariomycetes). Fungal Divers..

[40] Wu S.-H., Chen Y.-W., Shao S.-C., Wang L.-D., Li Z.-Y., Yang L.-Y., Li S.-L., Huang R. (2008). Ten-Membered Lactones from *Phomopsis* sp., an Endophytic Fungus of *Azadirachta indica*. J. Nat. Prod..

[41] Spatafora J.W., Sung G.-H., Johnson D., Hesse C., O’Rourke B., Serdani M., Spotts R., Lutzoni F., Hofstetter V., Miadlikowska J. (2006). A five-gene phylogeny of *Pezizomycotina*. Mycologia.

[42] Kusari S., Verma V.C., Lamshoeft M., Spiteller M. (2012). An endophytic fungus from *Azadirachta indica* A. Juss. that produces azadirachtin. World J. Microbiol. Biotechnol..

[43] Schubert K., Groenewald J.Z., Braun U., Dijksterhuis J., Starink M., Hill C.F., Zalar P., de Hoog G.S., Crous P.W. (2007). Biodiversity in the *Cladosporium herbarum* complex (Davidiellaceae, Capnodiales), with standardisation of methods for *Cladosporium* taxonomy and diagnostics. Stud. Mycol..

[44] Crous P.W., Braun U., Schubert K., Groenewald J.Z. (2007). Delimiting *Cladosporium* from morphologically similar genera. Stud. Mycol..

[45] Wikee S., Lombard L., Nakashima C., Motohashi K., Chukeatirote E., Cheewangkoon R., McKenzie E.H.C., Hyde K.D., Crous P.W. (2013). A phylogenetic re-evaluation of *Phyllosticta* (Botryosphaeriales). Stud. Mycol..

[46] Hibbett D.S., Binder M., Bischoff J.F., Blackwell M., Cannon P.F., Eriksson O.E., Huhndorf S., James T., Kirk P.M., Lücking R., Lumbsch H.T. (2007). A higher-level phylogenetic classification of the Fungi. Mycol. Res..

[47] Chowdhary K., Kaushik N., Coloma A.G., Raimundo C.M. (2012). Endophytic fungi and their metabolites isolated from Indian medicinal plant. Phytochem. Rev..

[48] Prakash H.S., Ruma K., Shailasree S., Kumara K., Sampath S., Ramachandrappa N., Prakash H.S. (2011). Diversity of Fungal Endophytes from Two Endemic Tree Species *Artocarpus hirsutus* Lam. And *Vateria indica* Linn. of Western Ghats, India. World J. Agric. Sci..

[49] Manawasinghe I.S., Phillips A.J.L., Hyde K.D., Chethana K.W.T., Zhang W., Zhao W.S., Yan J.Y., Li X. (2016). Mycosphere Essays 14: Assessing the aggressiveness of plant pathogenic Botryosphaeriaceae. Mycosphere.

[50] Rajagopal K., Kathiravan G., Karthikeyan S. (2011). Extraction and characterization of melanin from *Phomopsis*: A phellophytic fungi Isolated from *Azadirachta indica* A. Juss. Afr. J. Microbiol. Res..

[51] Suryanarayanan T.S., Ravishankar J.P., Venkatesan G., Murali T.S. (2004). Characterization of the melanin pigment of a cosmopolitan fungal endophyte. Mycol. Res..

[52] Zaidi K.U., Mani A., Ali A.S., Ali S.A. (2013). Evaluation of Tyrosinase Producing Endophytic Fungi from *Calotropis gigantea*, *Azadirachta indica*, *Ocimum tenuiflorum* and *Lantana camara*. Annu. Rev. Res. Biol..

[53] Selinheimo E., Saloheimo M., Ahola E., Westerholm-Parvinen A., Kalkkinen N., Buchert J., Kruus K. (2006). Production and characterization of a secreted, C-terminally processed tyrosinase from the filamentous fungus *Trichoderma reesei*. FEBS J..

[54] Abubakar S., Ndana R.W., Afolabi A.S. (2017). Bioprospective potentials of endophytic fungi *Penicillium spp.* isolated from leaves of *Azadirachta indica* (A. JUSS). Int. J. Biol. Res..

[55] Zhao J., Fu Y., Luo M., Zu Y., Wang W., Zhao C., Gu C. (2012). Endophytic fungi from pigeon pea [*Cajanus cajan* (L.) Millsp.] produce antioxidant cajaninstilbene acid. J. Agric. Food Chem..

[56] Wu S.-H., Chen Y.-W., Shao S.-C., Wang L.-D., Yu Y., Li Z.-Y., Yang L.-Y., Li S.-L., Huang R. (2009). Two New Solanapyrone Analogues from the Endophytic Fungus *Nigrospora* sp. YB-141 of *Azadirachta indica*. Chem. Biodivers..

[57] Verma V., Gond S., Mishra A., Kumar A., Kharwar R. (2008). Selection of Natural Strains of Fungal Endophytes from *Azadirachta indica* A. Juss, with Anti-Microbial Activity Against Dermatophytes. Curr. Bioact. Compd..

[58] Tan Q., Yan X., Lin X., Huang Y., Zheng Z., Song S., Lu C., Shen Y. (2007). Chemical Constituents of the Endophytic Fungal Strain *Phomopsis* sp. NXZ-05 of Camptotheca acuminata. Helv. Chim. Acta.

[59] Wu S.-H., He J., Li X.-N., Huang R., Song F., Chen Y.-W., Miao C.-P. (2014). Guaiane sesquiterpenes and isopimarane diterpenes from an endophytic fungus *Xylaria* sp.. Phytochemistry.

[60] Kaur H.P., Singh B., Thakur A., Kaur A., Kaur S. (2015). Studies on immunomodulatory effect of endophytic fungus *Alternaria alternata* on *Spodoptera litura*. J. Asia. Pac. Entomol..

[61] Verma V.C., Gangwar M., Yashpal M., Nath G. (2013). Anticestodal activity of endophytic *Pestalotiopsis* sp. on protoscoleces of hydatid cyst *Echinococcus granulosus*. Biomed Res. Int..

